# Dog Bites and Rabies in the Eastern Region of Ghana in 2013–2015: A Call for a One-Health Approach

**DOI:** 10.1155/2018/6139013

**Published:** 2018-07-02

**Authors:** Boakye-Yiadom Adomako, Frank Baiden, Samuel Sackey, Donne Kofi Ameme, Fred Wurapa, Kofi Mensah Nyarko, Ernest Kenu, Edwin Afari

**Affiliations:** ^1^Ghana Field Epidemiology and Laboratory Training Program (GFELTP), School of Public Health, University of Ghana, Ghana; ^2^Faculty of Infectious and Tropical Diseases, London School of Hygiene and Tropical Medicine, Keppel Street, London WC1E 7HT, UK

## Abstract

**Background:**

A One-Health approach is advocated to ensure effective rabies surveillance in sub-Saharan Africa. Information is needed to assess the current state of dog bites and rabies in Ghana. We analyzed data on reported events in the Eastern Region of Ghana from 2013 to 2015 to generate information that can be used for rabies elimination in Ghana through the One-Health approach.

**Method:**

We extracted data on dog bites and rabies from the database of the regional health service and performed descriptive analysis using Epi Info version 7™. We followed up with interviews with three key informants from the health and veterinary services on issues related to surveillance and data quality.

**Results:**

Overall, 4821 dog bites were reported over the three-year period. This translated into an annual incidence of 172 cases per a population of 100,000. Most of cases were in children aged less than 10 yrs. Fifteen (53.3% males) cases of rabies were recorded in seven out of the 26 municipalities and districts, translating into a rabies to dog bite ratio of 3: 1000. The median age of victims was 9 years (range: 3-72 years). A parallel and uncoordinated system of rabies surveillance is maintained by the health and veterinary services, with gross disparities in the number of reported events and overall impression of underreporting.

**Conclusion:**

Rabies remains an important cause of preventable deaths in this region. An integrated approach to surveillance based on the One-Health concept needs to be adopted.

## 1. Introduction

Rabies is a highly fatal neglected tropical disease that causes up to 59,000 deaths worldwide every year [1]. These deaths occur despite the availability of cost-effective preventive interventions that include timely and appropriate first aid wound management and postexposure prophylactic immunization (PEP). Ninety-nine percent of rabies cases result from the bite of rabid dogs [2–4]. The World Health Organization, the World Organization for Animal Health (OIE), the Food and Agriculture Organization, and the Global Alliance for Rabies Control (GARC) have established “United Against Rabies”, as a global collaborative program that is working towards achieving the goal of “zero human rabies deaths by 2030” [1, 5]. Achieving a good and effective surveillance system for rabies in endemic countries is a major intervention in the strategic plan of the Alliance.

Rabies is almost always fatal once symptoms begin. This makes primary prevention an imperative. Treating a rabies exposure can however have catastrophic financial burden on affected families. The average cost of rabies postexposure prophylaxis (PEP) is US$ 40 in Africa and US$ 49 in Asia. In the poor rural communities in Africa and Asia, where 99% of rabies deaths occur, average daily incomes are just about US$ 1–2 per person [6]. This makes PEP a challenging intervention to sustain in resource-poor settings. The mass vaccination of dogs is therefore an important strategy in rabies prevention. In many settings it has been found to be cost-effective and, through this approach, much of the developed world has eliminated rabies [6, 7]. In much of sub-Saharan Africa, imposition of user cost and lack of prioritization have contributed to unsustained programs of mass dog vaccinations in many countries.

Rabies is endemic in Ghana and it poses a major public health problem. Between 2000 and 2004, 123 clinically confirmed human rabies cases were reported by public health facilities across the country and between 2010 and 2014, 22 cases were seen at the Korle-Bu Teaching Hospital in Accra, the national capital [8].

The program to eliminate rabies is managed by two agencies. One of these is the Ghana Health Service (GHS) which is mainly responsible for the clinical care of suspected and confirmed cases. The elimination of rabies is part of the program on Neglected Tropical Diseases (NTDs) within the Ghana Health Service. The other agency responsible for rabies elimination in Ghana is the Ministry of Food and Agriculture (MOFA), acting through its Veterinary Services Division (VSD). This division is responsible for dog vaccinations, and the prevention and management of rabies in dogs and other animals. The lack of reliable data and systematic analysis of available data continues to keep rabies as a neglected condition in Ghanaian society. It remains a grossly underreported disease and there is very little systematically collected and analyzed data on dog bites and rabies, a major reason why the disease is neglected [9, 10].

We reviewed available data on dog bites and rabies in the Eastern Region of Ghana from 2013 through 2015 with the view of generating information that can be used for public health advocacy towards rabies elimination in Ghana, and for the One-Health approach to the control and elimination of zoonotic diseases [11, 12]. The specific objectives were to characterize cases of dog bites and rabies and to explore the current state of rabies surveillance, with specific reference to data quality.

## 2. Method

### 2.1. The Study Area

**Figure 1:**
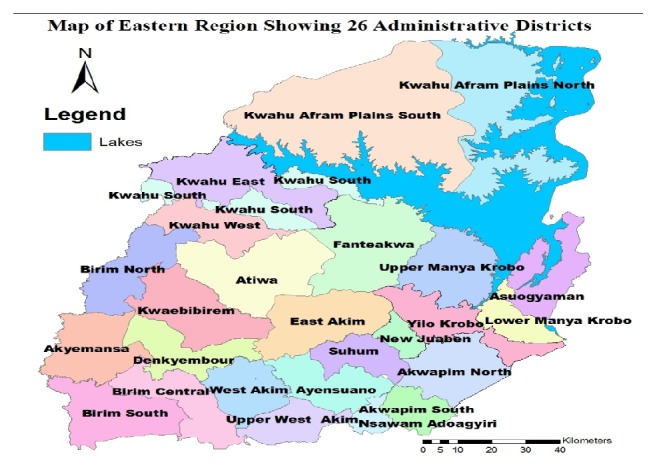
Map of Eastern Region showing 26 administrative districts.

The Eastern Region of Ghana lies between latitude 6.0°N and 7.0°N– and occupies a land area of 19,323km^2^, equivalent to 8% of the total land mass of Ghana. Sixty percent of the region is made up of tropical forest with the remaining 40% predominantly being Guinea Savannah. With an annual population growth rate of 2.1%, the region had a projected population of 2,861,405 as at 2014. It has 26 administrative districts and municipalities, with Koforidua being the regional capital [13] (Figure 1).

Cases of dog bites and rabies in animals in the Eastern Region are captured through the parallel surveillance systems of the GHS and the Regional Veterinary Unit (RVU) of the Ministry of Food and Agriculture (MOFA). It is generally recognized that by far most incidents are captured within the surveillance system of the GHS. The analysis presented in paper is based on events captured by the GHS and entered into its District Health Information Management System (DHIMS). A check was performed to obtain the number of cases that have been reported to RVU in Koforidua in the New Juaben Municipality.

### 2.2. Data Processing and Analysis

Data on reported incidents of dog bites and human rabies in the region from 2013 to 2015 were extracted from the DHIMS. A dog bite was defined as a bite inflicted upon a person by a dog resulting in wounds or scratches on the skin. Operationally all cases of dog bites are suspected cases of rabies. A confirmed case of rabies was defined as a suspected case with clinical and or laboratory confirmation. The clinical confirmation of rabies was based on a history of dog bite that is followed by classical symptoms such as anxiety, agitation, paralysis, excessive salivation, and hydrophobia.

Extracted data was entered and analyzed in Epi Info version 7. The distribution per districts as well as the age and sex distribution of cases was analyzed. The number of cases of dog bites and human rabies were converted into incidence (cases/100,000) based on 2014 population size projected from the 2010 Ghana population and housing census.

Two semistructured interviews were conducted with the heads of disease control and health information at the regional office of GHS, and the head of RVU in Koforidua. The object of the interviews was to explain the nature of the surveillance system and issues related to the quality of data. The responses provided are reported alongside the presentation of quantitative results.

### 2.3. Ethical Approval

Approval for this study was obtained from the Ghana Field Epidemiology and Laboratory Training Programme (GFELTP) under the School of Public Health at the University of Ghana. Administrative approval was also obtained from the Eastern Regional Health Directorate of the GHS. All extracted data was anonymized and did not have any individual identifiers. Only authorized persons had access to the data which was held in password-protected computers.

## 3. Results

**Figure 2:**
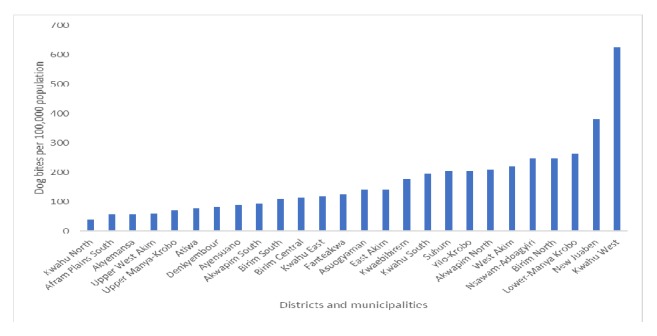
Dog bites per 100,000 persons in districts and municipalities in the Eastern Region of Ghana 2013-2015.

**Figure 3:**
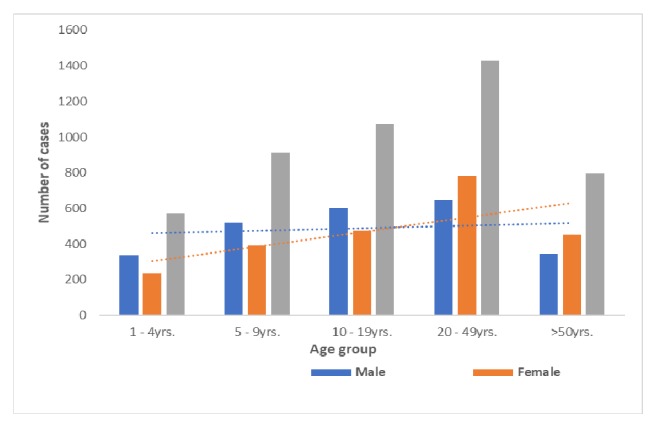
Cases of dog bites by age and sex.

A total of 4821 dog bites were reported over the three-year period. They were distributed as follows: 26 cases in 2013, 2360 cases in 2014, and 2435 cases in 2015. This translated into an annual incidence of 172 cases per 100,000 population. Most of the cases were in children aged below 10 yrs. The proportion of females increased from between 42.2% among children less than 10 years of age to 56.7% among adults aged over 50 yrs. The Kwahu West District recorded the highest (625 cases per a population of 100,000) cumulative incidence of cases followed by New Juaben Municipality, i.e., areas around the regional capital (380 cases per a population of 100,000). The Kwahu North district recorded the lowest (39 cases per a population of 100,000) incidence of cases (Figure 2). The trend was towards more women getting bitten by dogs (Figure 3).

During the follow-up interviews, it was explained that the wide disparity between the number of dog bites reported in 2013 and the numbers reported in 2014-2015 was due to a change in the data capturing system by the GHS. The DHIMS was introduced in 2012 as a new electronic disease data capturing platform. The entry of dog bites and rabies cases into the DHIMS however began in 2013. It was further explained that while the rabies surveillance system was facility-based and captured all cases reported to health facilities across the entire region, the surveillance system of the RVU was self-reporting and community-based but limited to Koforidua in the New Juaben Municipality. This accounts for the finding that DHIMS had 744 cases of dog bites reported for the New Juaben Municipality, while the RVU recorded only 136 cases. The GHS and RVU operated independent systems of zoonotic disease reporting, collected markedly different sets of information, and had no platform for data reconciliation.

### 3.1. Rabies

**Figure 4:**
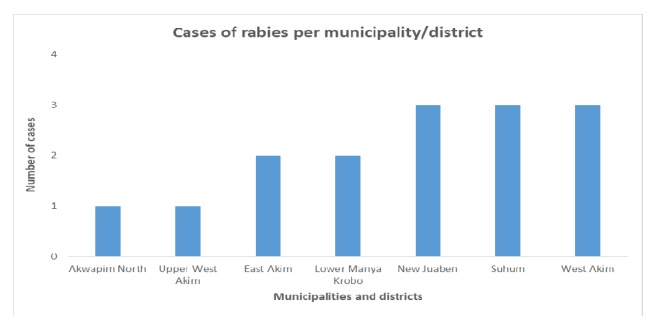
Cases of rabies per municipalities/districts in 2013-2015.

A total of 15 cases of rabies were reported into the DHIMS for the region from 2013 to 2015. Five, seven, and three cases were reported in 2013, 2014, and 2015, respectively. These cases were reported in seven districts as follows: three each in New Juaben Municipality, Suhum, and West Akim, two each in Lower Manya Krobo and East Akim, and one each in Akwapim North and Upper West Akim (Figure 4). Most (73%) of the cases were reported from urban communities in the respective districts. The average and median ages of victims were 20.0 years (standard deviation=23.4 years) and 9 yrs (range of 3-72 yrs), respectively, and females were nearly as equal (7 out of 15, i.e., 46.7%) as males. The trend was towards more females getting rabies with increasing age.

On a regional basis, the number of cases translates into a rabies to dog bite ratio of 3:1000. No data was available at either the GHS or the VSD about the population of dogs per district in the region. The average duration between dog bite and onset of rabies symptoms was 6 weeks with the longest being 16 and the shortest being 1 week. In the 82% of cases where data was available, no postexposure prophylaxis (PEP) was administered. The fatality rate was 100%.

## 4. Discussion

The key to understanding the epidemiology and burden of rabies lies in accurate and timely data. Poor and discrepant data underestimate the true burden of rabies and negate the advocacy efforts needed to achieve control and elimination [14]. This paper has presented a descriptive analysis of dog bites and rabies as reported through the DHIMS of the GHS in the Eastern Region of Ghana. Information provided by key officers of the GHS and the RVU have also been used to explain some of the data inconsistencies and incompleteness. Overall the findings reflect an uncoordinated state of rabies control in the region that manifest in poor surveillance, an underestimation of the risk of rabies in the region, and inadequacy in postexposure management of dog bites. The findings are consistent with reports from studies in other parts of Ghana and the subregion [9, 10, 15]. The problem of stray dogs, dog bites, and risk of rabies has persisted in Ghana for many years [16, 17].

The One-Health agenda currently championed by the Food and Agriculture Organization (FAO), the World Health Organization (WHO), and the World Organization for Animal Health (OIE) is a recognition of the fact that the control of zoonotic diseases such as rabies requires interdisciplinary and intersectoral collaboration. Over a decade and half of its adoption, there does not appear to be much in practical application in rabies surveillance in this region of Ghana [12]. The efforts in Ghana to eliminate rabies and other zoonotic diseases remain undermined by the lack of reliable data and coordinated, community-based surveillance. This problem extends to the global level where disparities in the number of rabies cases reported to WHO and the World Organization for Animal Health (OIE) continue to persist. Harmonizing the surveillance systems for zoonotic diseases as part of the One-Health agenda should be an important task within the Global Health Security program [11, 18]. Although very few examples of subnational-level implementation of the One-Health approach have been reported in sub-Saharan Africa, its effectiveness in improving disease prediction and control has been well-demonstrated in other parts of the world [19].

The finding that only three out of a 1000 cases of dog bites ultimately turned out to be rabid has important implications for the clinical management of cases of dog bites in resource-limited setting. By the protocol for the management of dog bites (suspected rabies cases), the dog is required to be put under observation for signs of rabies or PEP initiated immediately on the basis of a risk assessment that takes into consideration the local endemicity of rabies and what is known about the dog [20]. In most communities in Ghana dogs stray and it is barely possible to achieve effective quarantine or humane killing and laboratory confirmation as most protocols demand. In the circumstances, and where available, clinicians initiate presumptive postexposure prophylaxis once a report of dog bite is made and the dog cannot be traced, with a low prior probability of rabies this leads to wasteful use of highly expensive rabies vaccines. This was well-demonstrated in the city of N'Djaména in Chad where, following mass dog vaccination, the monthly animal rabies incidence dropped from 1.1/10,000 dogs to 0.061/10,000 dogs in 2014, and yet the demand for PEP remained largely unaffected [21, 22]. The path to rabies prevention following a dog bite is thus an arduous one for the affected person, the attending health worker, and the public health system. In the present study, in all 82% of cases for which data was available, no PEP was given due to nonavailability and cost. Until improved approaches to PEP that are currently being evaluated are confirmed, the best option for rabies prevention in low and middle-income countries like Ghana will remain mass dog vaccination and an integrated approach to risk assessment during case management [4, 23].

Our findings indicate that children aged less than 10 years were the most affected. This can be explained by the fact that children tend to be curious and have inadequate knowledge about dog behavior. Although overall we found more men (51.2%) to be bitten by dogs than women, with age, we observed a consistent, progressive, and disproportionate increase in the number of women bitten. A similar trend was reported in studies in Nigeria and South Africa and it may be due to increased dog ownership and/or outdoor activities by older women [24, 25]. It may also reflect differences in health-seeking behavior. The overall higher number of males is consistent with findings in studies in other parts of sub-Saharan Africa [26].

New Juaben Municipality recorded the highest number of rabies cases followed by Suhum and West Akim districts. All three districts had an incidence of dog bites of over 200 per a population of 100,000. Kwahu West had the highest incidence of dog bites recorded for the period under review (over 600 dog bites/population of 100,000). Apart from Upper West Akim, all the districts that reported cases of rabies recorded a dog bite incidence of over 100 per 100,000 persons. Due to a lack of data on the number of dogs in districts in Ghana, it is not proper to conclude that the number of dog bites could be related to population of dogs in the community. Despite the high incidence of dog bites, Kwahu West recorded no cases of rabies. This could be due to a coverage of vaccinated dogs or elimination of rabies virus from the district. The pattern suggests that the risk of rabies in the Eastern Region is focal with virus circulation occurring in specific locations. A well-coordinated effort at public education, mass dog vaccination, and strategic deployment of PEP should lead to early elimination of rabies in the region.

## Data Availability

The data that form the basis of this paper is available upon reasonable request to the corresponding author and /or Ghana Health Service.
